# Inferring Population Genetic Structure in Widely and Continuously Distributed Carnivores: The Stone Marten (*Martes foina*) as a Case Study

**DOI:** 10.1371/journal.pone.0134257

**Published:** 2015-07-29

**Authors:** María Vergara, Mafalda P. Basto, María José Madeira, Benjamín J. Gómez-Moliner, Margarida Santos-Reis, Carlos Fernandes, Aritz Ruiz-González

**Affiliations:** 1 Department of Zoology and Animal Cell Biology, Zoology Laboratory, University of the Basque Country (UPV/EHU), Vitoria-Gasteiz, Spain; 2 Systematics, Biogeography and Population Dynamics Research Group, Lascaray Research Center, University of the Basque Country (UPV/EHU), Vitoria-Gasteiz, Spain; 3 CE3C—Centre for Ecology, Evolution and Environmental Changes, Faculdade de Ciências, Universidade de Lisboa, Lisboa, Portugal; Smithsonian Conservation Biology Institute, UNITED STATES

## Abstract

The stone marten is a widely distributed mustelid in the Palaearctic region that exhibits variable habitat preferences in different parts of its range. The species is a Holocene immigrant from southwest Asia which, according to fossil remains, followed the expansion of the Neolithic farming cultures into Europe and possibly colonized the Iberian Peninsula during the Early Neolithic (ca. 7,000 years BP). However, the population genetic structure and historical biogeography of this generalist carnivore remains essentially unknown. In this study we have combined mitochondrial DNA (mtDNA) sequencing (621 bp) and microsatellite genotyping (23 polymorphic markers) to infer the population genetic structure of the stone marten within the Iberian Peninsula. The mtDNA data revealed low haplotype and nucleotide diversities and a lack of phylogeographic structure, most likely due to a recent colonization of the Iberian Peninsula by a few mtDNA lineages during the Early Neolithic. The microsatellite data set was analysed with a) spatial and non-spatial Bayesian individual-based clustering (IBC) approaches (STRUCTURE, TESS, BAPS and GENELAND), and b) multivariate methods [discriminant analysis of principal components (DAPC) and spatial principal component analysis (sPCA)]. Additionally, because isolation by distance (IBD) is a common spatial genetic pattern in mobile and continuously distributed species and it may represent a challenge to the performance of the above methods, the microsatellite data set was tested for its presence. Overall, the genetic structure of the stone marten in the Iberian Peninsula was characterized by a NE-SW spatial pattern of IBD, and this may explain the observed disagreement between clustering solutions obtained by the different IBC methods. However, there was significant indication for contemporary genetic structuring, albeit weak, into at least three different subpopulations. The detected subdivision could be attributed to the influence of the rivers Ebro, Tagus and Guadiana, suggesting that main watercourses in the Iberian Peninsula may act as semi-permeable barriers to gene flow in stone martens. To our knowledge, this is the first phylogeographic and population genetic study of the species at a broad regional scale. We also wanted to make the case for the importance and benefits of using and comparing multiple different clustering and multivariate methods in spatial genetic analyses of mobile and continuously distributed species.

## Introduction

In continuous populations, genetic structure vary from a single identifiable group of individuals, with gene flow only restricted by the distance among them, to an unknown number of subpopulations separated by barriers to gene exchange [1,2]. Species with high dispersal abilities and weak habitat specificity are expected to show little population genetic differentiation (e.g. [3]). However, recent studies conducted on broadly distributed species have revealed cryptic patterns of genetic structuring in apparently continuous populations due to historical processes and/or the presence of major topographic and landscape features [4–6]. Population and landscape genetic studies often apply individual-based Bayesian clustering (IBC) algorithms to detect genetic discontinuities, which are subsequently correlated with landscape characteristics [1,7–10]. The application of IBC methods to data sets characterized by a pattern of isolation by distance (IBD) may, however, often result in an overestimation of population subdivision, especially when the sampling design is irregular [1,11,12]. Thus, [13] suggested comparing the outputs of several IBC algorithms, in addition to testing for the presence of IBD, to infer a genetic structuring that could be empirically explained. The performance of IBC methods in landscape genetics has been extensively demonstrated in studies and simulations comparing different approaches [1,8,10,11,14–17]. STRUCTURE is one of the most widely used IBC algorithms to infer the number of clusters in a data set and assign individuals to those clusters based exclusively on their multilocus genotypes [18,19]. Conversely, IBC programs such as BAPS [20], GENELAND [14] or TESS [21,22], allow the use of geo-referenced samples in their spatially-explicit models. The spatial prior (i.e. the geographic location of each sample) may provide more support to clustering solutions and be particularly helpful in the case of sparse sampling [23]. Multivariate ordination analyses [e.g. discriminant analysis of principal components (DAPC) and spatial principal component analysis (sPCA)]) have been suggested as an alternative to IBC algorithms because they do not make any assumption about the underlying population genetic model [24,25]. Thus, a consensual solution from the combination of different approaches (i.e. Bayesian and multivariate analyses) can be used to obtain reliable inferences on spatial genetic patterns [10,15,17], which may be particularly cryptic and/or complex in mobile and continuously distributed taxa [3].

The stone marten, (*Martes foina*, Erxleben, 1777) is one of the most widely distributed mustelids in Eurasia [26]. It is present in the Middle East, central Asia and in central and southern Europe, with the Iberian Peninsula representing the western limit of its distribution. In parts of its range the stone marten is associated with human-dominated environments (e.g. [27]) but in Iberia the species prefers mosaic habitats [28], forest, scrublands and rocky areas rather than urbanized environments [29–31]. Nevertheless, its generalist ecological requirements contribute to the species being locally abundant and ubiquitous in the Iberian Peninsula. The limited fossil evidence suggests that stone martens are present in this region since the middle Holocene (*ca*. 7,000 years before present (BP) [32,33]), as a result of a range expansion by the species from southwest Asia into Europe early in the Holocene following the expansion of the Neolithic farming cultures of the Near East [32–34]. However, the details of the historical biogeography of the stone marten in Europe are essentially unknown. In any case, the Iberian Peninsula, because it is only connected to the rest of continental Europe by the Pyrenean isthmus, offers an interesting scenario to investigate how landscape barriers shape the distribution and population structure of the species as it expanded throughout the peninsula after its arrival in Iberia.

Several genetic studies within *Martes* species with a phylogeographic or taxonomic focus have relied on mtDNA sequencing (mainly D-loop and Cytochrome *b* regions) because the relatively fast evolutionary rate of mtDNA is well suited to examine events that occurred hundreds of thousands to a few million years ago (reviewed in [35]). More recently, the number of studies on *Martes* spp. from population or conservation genetic perspectives and using microsatellites has steadily increased [6,36,37]. In contrast, either phylogeographic or population and landscape genetic investigations on the stone marten are virtually absent from literature [35], with the exception of two country-level studies [38,39]. Research on the genetic structure of *M*. *foina* should be particularly interesting and challenging because it is more generalist than many other marten species [40].

In this study, we use mtDNA and microsatellite markers to investigate the phylogeography and genetic structure of *M*. *foina* in the Iberian Peninsula. This is the first thorough phylogeographic and population genetic survey of this species at a broad regional scale. The results of this work shed light on both the species’ history and current population structure in Iberia. We also use this case study to illustrate and argue for a consensual approach comparing complementary methods with distinct assumptions (Bayesian clustering algorithms and multivariate analyses) to accurately characterize genetic structure and to identify processes shaping gene flow.

## Materials and Methods

### Ethic Statement

Samples included in the present study (S1 Table) were obtained from different sources; a) specimens from museum collections (n = 68), b) samples from Wildlife Rescue Centres (WRC) collected by veterinaries with the permission of the corresponding regional wildlife authority (n = 77), c) samples from road-killed individuals collected by wildlife researchers, veterinaries and rangers (n = 154) and additionally, d) hair samples from live-trapped individuals obtained in the framework of field studies for other purpose than this study (n = 53). Further details for each sample regarding its origin, type of collection, collector and permission obtained (when requested) are listed in S1 Table. No animals were sacrificed or captured for this study. Therefore, a specific formal approval by an Institutional Animal Care and Use Committee was not necessary.

### Study area

**Fig 1:**
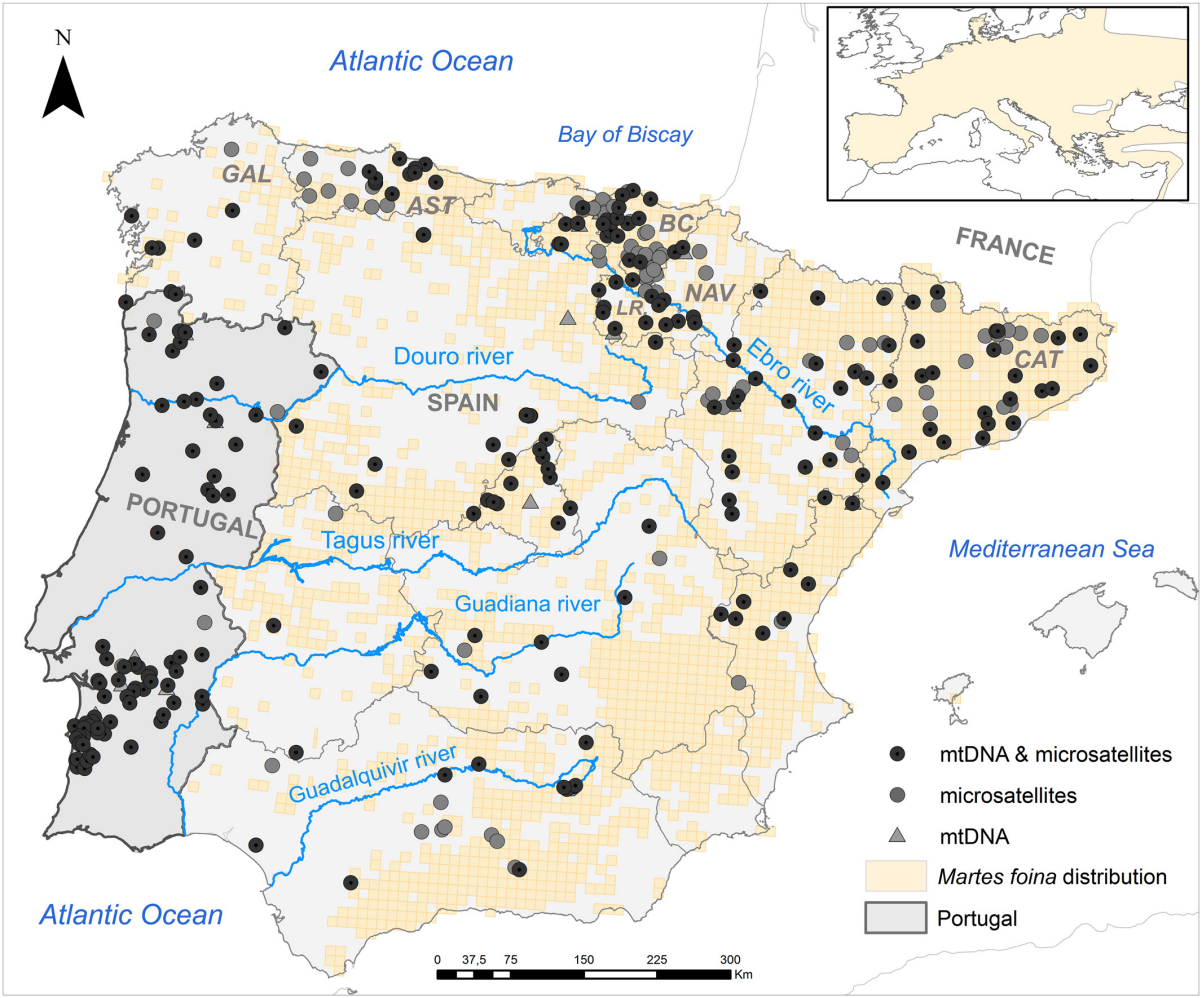
Map of the study area and of the stone marten distribution in Spain [41] with the geographic location of each genotyped (microsatellites) and/or sequenced (mtDNA) sample in the Iberian Peninsula. The inset shows the species range in Europe [26]. Dark grey dots, light grey dots and grey triangles represent individuals with microsatellite and mtDNA data, only microsatellite data and only mtDNA data, respectively. The Autonomous Communities mentioned in the paper are coded as follows: Galicia (GAL), Asturias (AST), Basque Country (BC), La Rioja (LR), Navarre (NAV) and Catalonia (CAT). The distributions maps were specifically generated based on the information provided in [41] and [26] using the country borders and the reference grids layers from the European Environmental Agency as background, available in http://www.eea.europa.eu/data-and-maps/ under a Creative Commons Attribution license, and are therefore for illustrative purposes only.

The study area was the Iberian Peninsula, a region of 582,000 km^2^ at the south-western edge of Europe and comprising the mainland territories of Spain and Portugal and the country of Andorra (Fig 1). On the north, west and southwest it is bordered by the Atlantic Ocean and on the east and southeast by the Mediterranean Sea, while the Pyrenees form the northeast edge of the peninsula, separating it from the rest of Europe. Iberia has a relatively high average altitude (600 m) due to the presence at the centre of a vast plateau, known as the Meseta. A series of other mountain ranges around the plateau and others located on the periphery of the peninsula complete the topographic outline. The major rivers, Ebro, Douro, Tagus, Guadiana and Guadalquivir, flow through the wide valleys between mountain systems. The Iberian Peninsula has the most varied mosaic of climates in Europe and represents the western limit of the stone marten distribution ([26], Fig 1).

### Sample collection and DNA extraction

A total of 599 tissue and hair samples (Spain: n = 509; Portugal: n = 90) were obtained from museum collections and wildlife rescue centres or collected from road-killed or live-trapped stone martens throughout the Iberian Peninsula (Fig 1, S1 Table). Tissue samples were preserved in ethanol 96% and frozen at -20°C, while hair samples were stored in dry tubes until DNA extraction. To monitor potential contaminations, we included one negative extraction control per extraction session. DNA was extracted with the Qiagen DNeasy Tissue kit following the manufacturer’s protocol for tissue and hair samples.

### Mitochondrial DNA sequencing and microsatellite genotyping

A fragment of 621 bp of mtDNA encompassing a portion of the cytochrome *b* gene, the threonine and proline tRNAs and the left domain of the control region was amplified with the primers L15533 [42] and H16437 (5′- GGA GCG AGA AGA GGT ACA-3′). PCRs were carried out in 15 μL comprising 3 μL of DNA extract (20–100 ng), 1X PCR buffer (NZYTech), 2 mM MgCl2, 0.2 mM of each dNTP (Bioline), 1 uM of each primer, 0.17 ug/uL BSA (New England Biolabs) and 1.25U of Supreme NZYTaq DNA polymerase (NZYTech). The reactions were performed with an initial denaturation step at 94°C for 5 min, followed by 55 cycles at 94°C for 30 s, 60°C for 30 s and 72°C for 1 min. The final extension was at 72°C for 7 min. PCR products were purified as in [42] and sequenced at Macrogen Inc. Electropherograms were visually inspected using SEQSCAPE 2.5 (Applied Biosystems) and nucleotide sequences were aligned using the default parameters in CLUSTALX 2.0 [43] and manually checked in BIOEDIT 5.0.9 [44].

Samples were individually genotyped with a novel multiplex panel of 23 autosomal microsatellite markers comprising 13 recently described species-specific microsatellites (Mf 1.1, Mf 1.11, Mf 1.18, Mf 1.3, Mf 2.13, Mf 3.2, Mf 3.7, Mf 4.10, Mf 4.17, Mf 6.5, Mf 8.7, Mf 8.8, Mf 8.10; [45]) and 10 additional markers described in closely related mustelids (Ma1 and Ma2 [46]; Mel1 [47]; MLUT27 [48]; Mvis072 [49]; Mvi57 [50]; Lut615 [51]; MP0059 [52]; and Lut435 and Lut453 [53]). The forward primers labelled with the dyes 6-FAM, NED, PET and VIC, were used in four PCR multiplex reactions (multiplex A, B, C and D; S2 Table). PCR multiplex amplifications were carried out using the QIAGEN Multiplex PCR kit, following the manufacturer’s protocol, at optimized annealing temperatures in a total volume of 9 μL with 1 μL of DNA and 2 pmol of each primer. We applied a hot-start thermocycling protocol with an initial polymerase activation at 95°C for 15 min, followed by 42 cycles of denaturation at 94°C for 30s, primer annealing at 57°C for 130s, and sequence extension at 72°C for 1 min, and a final extension step at 60°C for 30 min. In addition to the negative controls for extraction, negative PCR controls were included. Multiplex PCR products were run on an ABI 3130XL automated sequencer (Applied Biosystems) with the internal size standard GS500 LIZ (Applied Biosystems). Fragment analyses were performed using GENEMAPPER v. 4.0 (Applied Biosystems).

The software GIMLET v 1.3.4 [54] was used to calculate the probability of identity with both the unbiased equation for small sample size (PID) and the equation for siblings (PID-sibs), and to estimate genotyping errors [i.e. allelic dropout (ADO) and false alleles (FA)] from replicate genotyping. To check for genotyping errors in hair and tissue samples, we amplified 30 DNA extracts (hair samples: n = 20; tissue samples: n = 10) in four replicates. As errors were absent in both sample types, the remaining sample set was genotyped only once per locus.

To map the spatial distribution of the analysed samples in the study area, the UTM coordinates corresponding to each sample were projected onto a GIS (ArcGIS 10.0, ESRI) along with the haplotype and microsatellite data.

### Mitochondrial DNA analysis

ARLEQUIN v. 3.5 [55] was used to estimate the amount of mtDNA variation through haplotype diversity (H) and nucleotide diversity (π) [56]. Haplotypic richness (Hr), standardized to the lowest sample size, was calculated using the rarefaction method implemented in CONTRIB v. 1.02 [57]. To infer haplotype relationships, a median-joining network was constructed as implemented in NETWORK v. 4.6 [58]. The program DNAsp v. 5 [59] was used to calculate distributions of pairwise nucleotide differences (mismatch distributions), which may be informative about past demographic history: unimodal curves are expected in populations that have undergone a rapid population expansion and multimodal curves are typical of populations with a history of long-term demographic stability [60,61]. We also applied two neutrality tests in ARLEQUIN (Fu’s Fs, [62]; Tajima’s D, [63]) to obtain further clues concerning demographic history; significance was tested using 1,000 simulations under a model of selective neutrality.

Historical population size changes were also assessed using a coalescent-based Bayesian Skyline Plot (BSP) generated in BEAST v. 1.7.5 [64]. Under a coalescent model it is possible to infer population parameters from genetic sequence data, such as estimates of mutation rate, divergence time, and effective population size through time. A relaxed lognormal clock was selected to construct the BSP. A substitution rate (μ) of 1.95 x 10^−8^ substitutions per nucleotide per year was estimated using equation D = 2μT [56], where D is genetic distance and T is the time since divergence. Specifically, as an estimate of D we computed in MEGA v.6 [65] the raw maximum composite likelihood distance [66] between the stone marten haplotypes obtained in this study and the published haplotypes of sable *Martes zibellina* (Genbank Accession Nos. KJ202610, KJ202613-5, KJ202623, KJ202625-7, KJ202633, KJ202636, KJ202640, KJ202644), and we assumed a time to the most recent common ancestor of 2.8 million years [67,68]. The best-fit model of nucleotide substitution for these sequences was HKY+I+G (α = 0.33, I = 0.780) according to the Akaike Information Criterion in jModelTest2 [69]. Chains were run for 75 million generations; trees were sampled every 1,000 generations and the first 10% of the samples were discarded as burn-in. Four replicate runs were conducted to confirm convergence.

### Population structure: Bayesian clustering and multivariate analysis

Population structure was first estimated using the program STRUCTURE v. 2.3.4 [18,19,70] assuming population admixture and correlated allele frequencies within populations. Simulations were run with Markov Chain Monte Carlo (MCMC) of 10^6^ iterations after a burn-in of 10^5^. *K* was allowed to vary from 1 to 10 and 20 independent simulations were run for each *K* value to check for consistency in the results. To determine the most likely number of clusters we estimated the rate of change in the log probability of data between successive *K* values (ΔK) as described by [71]. For the identified *K* value, we used CLUMPP v. 1.1.2 [72] to determine the population assignment probability of each individual across all simulations. Subsequently, a progressive partitioning approach, forcing K = 2 within each inferred cluster, was used to test for finer sub-structuring (e.g. [8]). GENELAND v 4.0.3 [14] was run through an extension of R v.3.0.1 [73] under the correlated allele frequency model without spatial uncertainty in spatial locations. We allowed K to vary between 1 and 10 in 20 independent runs, each with 10^5^ iterations, a thinning of 100, a maximum number of nuclei of 1,000, and a maximum rate of Poisson process fixed at 333. The software TESS v.2.3.1 [21,22,74] is designed for seeking genetic discontinuities in continuous populations and estimating spatially varying individual admixture proportions, and can outperform other IBC methods at detecting migrants and recent contact zones between weakly differentiated populations [21]. Following the recommendations in [21], TESS runs were performed under the admixture model for 12,000 sweeps, with a burn-in period of 2,000 sweeps, interaction parameter set to 0.6, 10 independent runs per analysis and the maximum number of clusters fixed to Kmax = 10. Lastly, BAPS v. 6.0 [75] was run for both the microsatellite and mtDNA data sets using spatial clustering of individuals, since the spatial prior may strengthen inferences for sparse molecular data [23]. Then, the membership coefficients of each individual calculated by the different IBC programs were plotted on a map of the study area in ArcGIS 10.0 (ESRI), to assess the relationship between genetic discontinuities and landscape features.

Multivariate analyses have been suggested as an alternative to Bayesian clustering algorithms. Their main asset is that they can summarize the genetic variability without making strong assumptions about the underlying population genetic model, as they do not require populations to be in Hardy-Weinberg equilibrium (HWE) or linkage equilibrium (LE) between loci [24,25]. For instance, the discriminant analysis of principal components (DAPC), [25] identifies genetic clusters through sequential clustering and model selection. The method first transforms the genotype data into principal components and then uses k-means clustering to define groups of individuals, with the best-supported number of clusters identified by the Bayesian Information Criteria (BIC). The spatial principal component analysis (sPCA, [24]) can explicitly identify cryptic spatial patterns of genetic structuring across the landscape, including clines, accounting for spatial autocorrelation issues associated with neighbour-mating and sample distribution [11].

We tested for the presence of IBD across the study area using both a Mantel test and a spatial autocorrelation analysis. The Mantel test was performed between a matrix of pairwise Edward’s genetic distances [76] and a matrix of Euclidian geographic distances, with 9999 permutations to assess significance. The Mantel test, as well as DAPC and sPCA, were implemented in R software v.3.0.1 [73] with the adegenet package [24]. Spatial autocorrelation analysis, which allows examining the correlation of genetic and geographic distance at multiple distance classes, is typically more powerful than Mantel tests for uncovering genetic structure [77]. The spatial autocorrelation analysis was performed in GENALEX v 6.5 [78]. GENALEX generates an autocorrelation coefficient (*r)*, which provides a measurement of the pairwise genetic similarity of individuals whose geographic separation falls within a specified distance class. The significance of the resulting correlogram was determined with the heterogeneity test of [79].

Finally, we used the software ALLELES IN SPACE (AIS, [80]), specifically developed to deal with cases in which individuals are continuously distributed possibly over large spatial scales to obtain a genetic landscape shape interpolation (GLSI) that allows visualizing patterns of genetic diversity across the landscape (a 50x50 grid surface with the distance weighting parameter set at 1). We also used AIS to conduct an allelic aggregation index analysis (AAIA, [80]), a test of non-random patters of spatial genetic diversity. Significance of R_AVE_ (average allelic aggregation index) was tested through the use of 1,000 permutations.

### Genetic diversity over the Iberian Peninsula and within each inferred cluster

Microsatellite genetic variation was characterized, for the whole study area and each inferred cluster, by the number of alleles per locus (N_A_) and inbreeding coefficient (Fis) using GENETIX v 4.05 [81]. The software FSTAT v.2.9.3 [82] was used to calculate the observed and expected heterozygosities (H_O_ and H_E_), the allelic richness (A_R_), the deviations from HWE, the genotypic disequilibrium among pairs of loci, and pairwise Fst values [83]. Statistical significance was evaluated by running a MCMC consisting of 10,000 batches of 10,000 iterations each, with the first 10,000 iterations discarded before sampling [84]. Significance levels were adjusted with sequential Bonferroni correction in order to correct for the effect of multiple tests [85]. In addition to Fst, we also estimated genetic differentiation using Jost’s D_EST_ [86] and G''_ST_ [87] in GENALEX v 6.5 [78]. MICROCHECKER v.2.2 [88] was used to check for potential scoring errors and the presence of null alleles. Sibship analysis was conducted using COLONY v. 2.0.4 [89], with a typing error rate set at 0.01. This approach considers the likelihood of the entire pedigree, as opposed to relatedness of individuals on a pair-wise basis.

## Results

### Mitochondrial DNA

After alignment and trimming the ends, a total of 621 base pairs were obtained from the 252 sequenced individuals. Of the 621 aligned nucleotide sites, 12 were variable and 10 were parsimony informative, leading to a nucleotide diversity of 0.00169. We identified 12 haplotypes (H1–H12), 11 excluding sites with gaps, and haplotype diversity was therefore relatively low (0.705). Sequences of haplotypes H1 to H12 were deposited in Genbank (Accession Nos. KM972577-KM972588, S1 Table).

**Fig 2:**
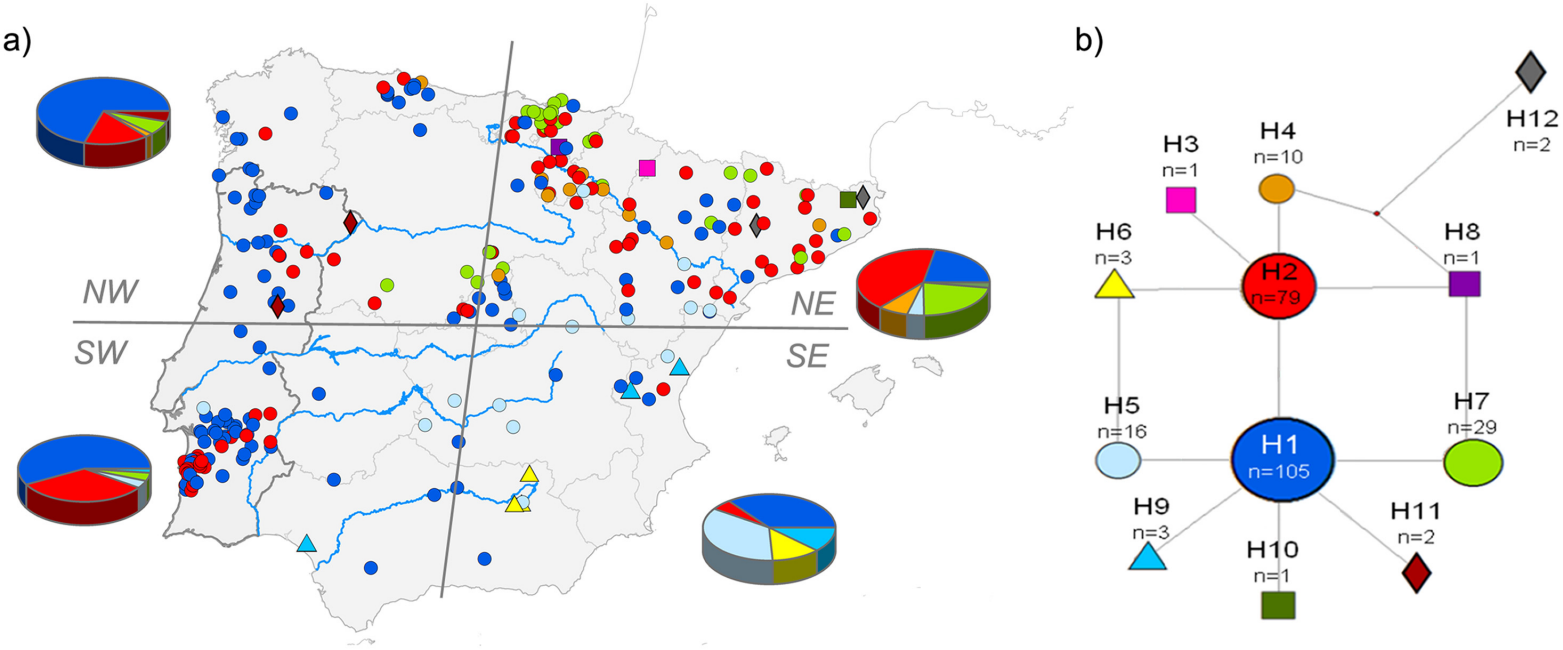
a) Geographic location of the 252 individuals sequenced for mtDNA in the Iberian Peninsula, which was divided into four regions (NE, NW, SE and SW). Pie charts represent the proportion of the samples with each haplotype in each region. **b**) Median-joining network of the 252 mtDNA sequences. Each haplotype (H1 to H12) is represented by a different coloured symbol (the same in the map). Grey numbers on the lines connecting haplotypes indicate the position of mutations in the alignment. “n” is the number of sequences in each haplotype. Less frequent haplotypes are represented by a square (n = 1), diamond (n = 2) and triangle (n = 3) to facilitate their identification.

The median-joining network (Fig 2) showed a star-like topology with most haplotypes differing by a single mutation from two common central haplotypes (H1 and H2), themselves also separated by a single substitution. Haplotype H12, found only in two samples from near the eastern Pyrenees, at eight mutational steps from the centre of the network, was the sole exception to this pattern. The network did not suggest any marked phylogeographic structure, with the most common haplotypes (H1: 41.6%; H2: 31.3%) scattered throughout the Iberian Peninsula. However, haplotype frequencies differed notably between regions, with all of them having private haplotypes with the exception of region SW (Fig 2). In the NW, H1 was the most common haplotype (68.5%) followed by H2 (20.4%), H7 (5.6%), the private haplotype H11 (3.7%) and H4 (1.8%). In the NE (Fig 2), H2 was the most frequent haplotype (38.9%), but some private haplotypes were also found in this region (H3, H8 and H10), including the most divergent haplotype H12, rendering the NE region the one with the highest haplotype diversity and haplotypic richness (S3 Table). The SW region, mostly corresponding to western Andalucía and south Portugal, harboured 5 haplotypes, the widespread H1 and H2 (55.9 and 36.8% respectively) plus H5, H7 and H9. Lastly, in the SE region, the analysed samples had haplotypes H1 (38.1%), H5 (33.3%), H9 (9.5%), H2 (4.8%) and the private haplotype H6 (14.3%).

The tests of Tajima´s D (-1.10943; p>0.1) and Fu´s Fs (-3.344; p>0.02) did not reject the null hypothesis of demographic stability. However, the BSP indicated a period of constant population size from 6,500 to 1,000 YBP, after which the Iberian population experienced a slow demographic expansion.

BAPS analysis highlighted the mtDNA homogeneity of Iberian stone martens, as it only identified two clusters: one grouping all samples with haplotypes H1–H11 and another exclusively with the two individuals carrying the divergent haplotype H12 that were sampled in NE Iberia close to the French border (Fig 2).

### Microsatellites

We genotyped 333 stone martens from across the Iberian Peninsula (Spain: n = 244; Portugal: n = 89) and we obtained full multilocus genotypes at the 23 loci for 257 of the samples (77.17%). All samples were genotyped for more than 20 loci and therefore included in the analyses.

The number of alleles ranged from four (Mf 1.11, Mf 1.18, Mf 2.13 and LUT453) to 13 (Mf 4.17) with a mean number of alleles per locus of 6.52 (S2 Table). The overall PID using all 23 loci was 3.563 X 10^−17^ and the overall PID-sib was 1.075 X 10^−7^. Mean observed heterozygosity over all loci (Ho = 0.49) was lower than expected heterozygosity (He = 0.57). Twenty-four rare alleles (frequency <0.05) were detected while very rare alleles (frequency <0.01) were not found.

To avoid effects due to the underlying genetic structure, some analyses and tests were performed within the inferred clusters (see next section). Linkage disequilibrium was not apparent for any pair of loci within any of the clusters, before or after Bonferroni correction (p<0.05). Fis values were positive and significantly different from zero (p<0.001, S4 Table). As recommended by [90], the presence of null alleles was tested in three sets of 25 individuals from South Portugal, Basque Country and Catalonia, based on the results of the clustering analyses, and only three out of the 23 amplified loci showed signs of null alleles (Ma-2, Mf 2.13 and Mf 4.10). COLONY identified 16 full-siblings sampled from less than 1 km away (two road-killed individuals in Burgos, Spain) to almost 70 km apart (two samples collected in Évora district, Portugal), whereas no parent-offspring dyads were detected. Thus, eight individuals (one from each dyad) were excluded from the dataset because the removal of full-siblings is expected to improve estimates of population structure [91].

### Population genetic structure

**Fig 3:**
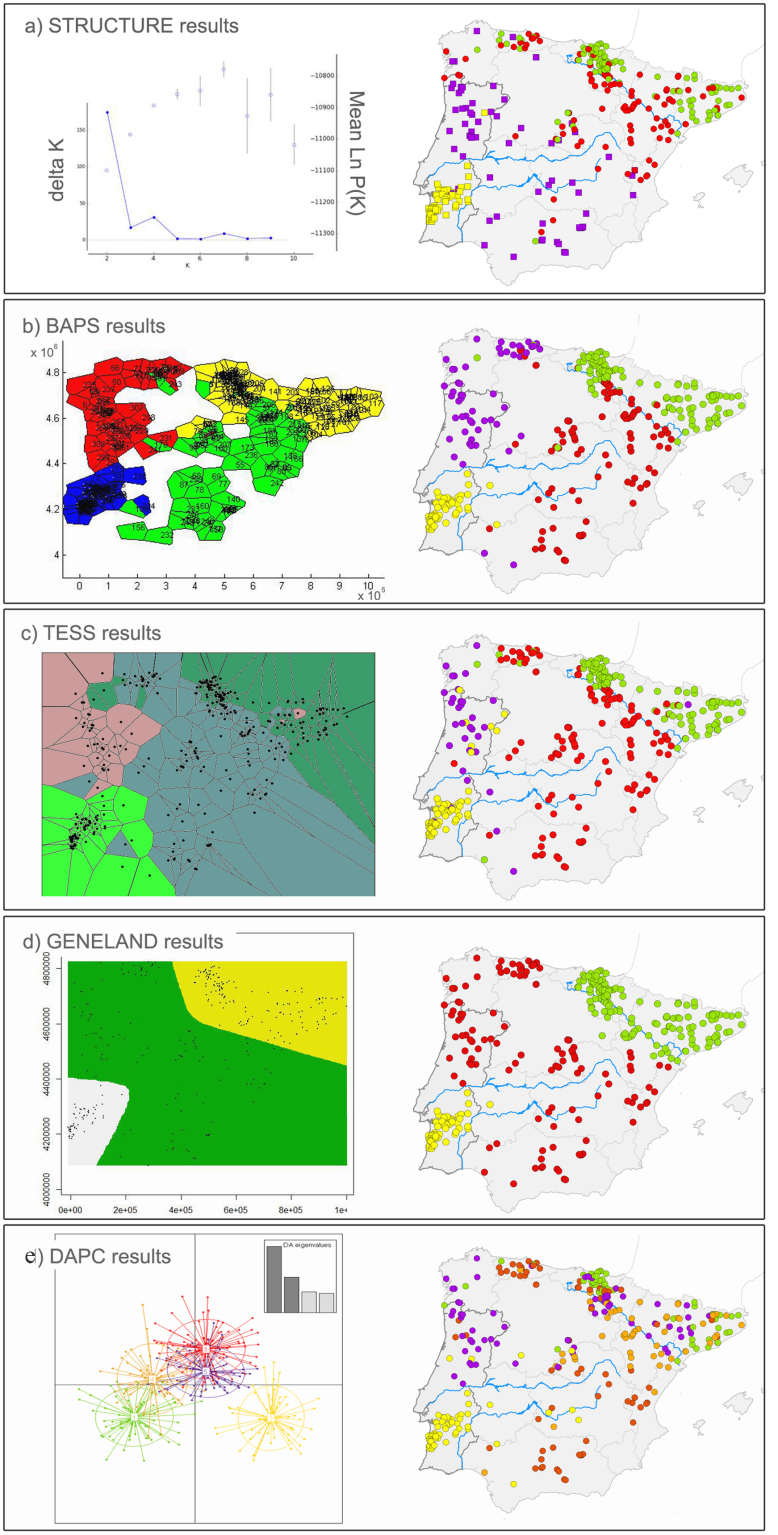
Map of the study area showing the results of the Bayesian clustering algorithms and the DAPC analysis. Individuals are represented by dots and coloured to reflect the cluster they were assigned to with the highest membership coefficient in each analysis. The Tagus, Ebro and Guadiana rivers are represented in blue. **a**) Plot from Structure Harvester for STRUCTURE results showing the modal value of K = 2 for the ΔK method (left axis; blue dots connected by line) and K = 4 for the maximum likelihood method (Mean L(K)± SD, right axis; open circles); in the latter case the chosen K is that after which L(K) plateaus or increases slightly and the variance between runs increases; **b**) BAPS results (K = 4); **c**) TESS results (K = 4); **d**) GENELAND results (K = 3);**e**) DAPC identified K = 5 as the optimal number of clusters, each indicated by different colours and inertia ellipses, the latter shown on the first two axes.

**Fig 4:**
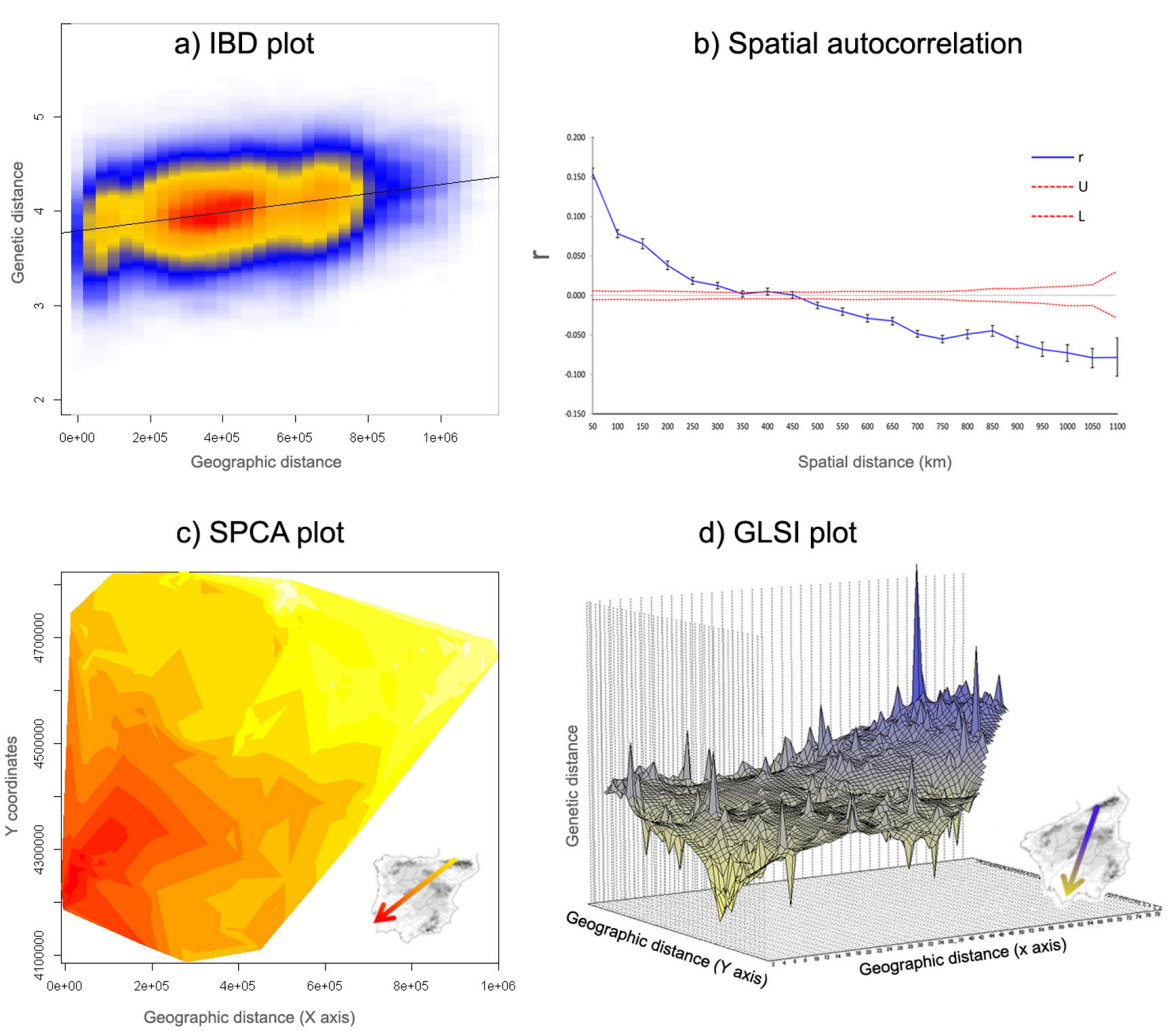
**a**) Scatterplot showing the results of the Mantel test between the matrix of genetic distances and the matrix of geographic distances to test for the presence of IBD. Colours represent the relative density of points, with warmer colours indicating higher densities, while the line shows the correlation between the two distance matrices. **b**) Correlogram of the average autocorrelation coefficient (r) as a function of distance classes of 50 km. Error bars bound the 95% confidence interval determined by bootstrap resampling (1,000 replicates) while confidence limits bound the 95% confidence interval about the null hypothesis of no spatial structure as determined by 999 permutations. **c**) Interpolation using a globally weighted regression of component 1 scores from the sPCA. Contours are component scores representing similarity across the landscape, with the arrow indicating the gradient found within the study area. **d**) Genetic Landscape Shape Interpolation (GLSI). Surface plot heights reflect genetic distance patterns over the geographical landscape examined and the arrow indicates the gradient found within the study area.

Among the Bayesian clustering approaches, the non-spatial algorithm implemented in STRUCTURE identified two clusters at the uppermost level (K = 2, represented by dots and squares in Fig 3a) following the criterion of Evanno et al. [71]. Individuals located at the NE and SW extremes had the highest membership coefficients (>0.9) while the other samples elsewhere in the study area showed admixed ancestry (data not shown), a pattern consistent with IBD structure (see below and also Fig 4a). Progressive partitioning suggested subdivision of the two clusters above, a clustering solution also hinted by the ΔK criterion. Of the four clusters, only one was relatively well delimited (in south Portugal, represented in yellow), but it was possible to roughly associate the others with NE Spain (Basque Country and Catalonia, in green), with an area from east to northwest Spain (in red), and with an area from north Portugal to south Spain (in purple) (Fig 3a). BAPS and TESS also suggested K = 4 but with different cluster compositions among each other and from progressive partitioning in STRUCTURE, especially for the two middle clusters between NE Spain and SW Portugal (Fig 3b and 3c).

BAPS grouped north Portugal with NW Spain (Galicia and Asturias) (in purple) and inferred a cluster in east and central Spain from south of the River Ebro down to Andalucía (in red) (Fig 3b), while TESS assigned individuals from Asturias to the latter group (in red; Fig 3c). GENELAND inferred the clustering solution (K = 3) with higher spatial consistency and more clearly defined cluster boundaries (Fig 3d). The three identified genetic units are distributed in south Portugal (in yellow, n = 57), over a large part of Iberia from NW Spain and north Portugal to east and south Spain (in red, n = 126), and in NE Spain (in green, n = 150).

Overall, the different Bayesian clustering methods converged in identifying two distinct genetic units in NE Spain and south Portugal (Fig 3). BAPS and TESS detected two additional clusters but they were incongruent between the two methods, while GENELAND was more conservative and grouped all the individuals elsewhere between NE Spain and south Portugal into a single cluster. The GENELAND solution was the most consistent and meaningful geographically, and so the GENELAND clusters were chosen for further analyses of genetic diversity (S5 Table) including IBD (see below).

The multivariate DAPC identified K = 5 as the optimal number of clusters by the Bayesian Information Criterion but, with the exception of the cluster in south Portugal, they were spatially admixed (Fig 3e). The first principal component differentiated cluster 2 (in yellow) from the others, and the second principal component displayed a slight differentiation between cluster 1 (in green, mainly found in NE Spain) and clusters 3, 4 and 5 (in red, orange and purple respectively, and scattered and overlapping over the majority of the study area). Except for cluster 2, the ellipses delineating the spatial extent of each cluster were substantially overlapping, suggesting weak genetic structuring overall.

Pairwise Fst values between clusters estimated by the different clustering methods ranged from 0.033 to 0.137 (S5 Table). Pairwise values of D_EST_ and G''_ST_, which correct the dependency of Fst on the amount of within-population variation, were moderate to large and statistically significant (S6 Table). Notably, there was no agreement between the Fst values and the degree of geographic separation or how clearly defined were the boundaries between clusters.

An overall pattern of IBD was detected across the study area by both the Mantel test (0.296, p < 0.001; Fig 4a) and the spatial autocorrelation analysis (heterogeneity test, p<0.01). The Mantel regression plot (Fig 4a) displayed a single high-density nucleus indicating clinal variation, while the autocorrelation correlogram (Fig 4b) showed a decline in the average relatedness as a function of linear geographic distance, with positive and significant values up to 450 km. The x-intercept of 452 km provided an estimate of the extent of non-random (positive) genetic structure, i.e., of the “genetic neighbourhood” of a population [92] (Fig 4b). The sPCA (r = 0.633 p<0.001; Fig 4c) and the GLSI from the AIS software (Fig 4d) revealed a cline in allele frequencies from NE to SW Iberia. The AAIA identified significant allelic aggregation (R_AVE_ = 0.735, P = 0.000), suggesting a non-random distribution of genotypes across the Iberian Peninsula. The strength of the spatial pattern along the NE-SW axis is further illustrated by the results of the clustering analyses (Fig 3).

## Discussion

### Phylogeography of the stone marten in the Iberian Peninsula

The results of several analyses of the mtDNA data support a relatively recent expansion of the stone marten population in the Iberian Peninsula, including the star-like topology of the median-joining network, the unimodal shape of the mismatch distribution, and the BSP estimates. The summary statistics Tajima´s D and Fu´s Fs did not reject the null hypothesis of selective neutrality and demographic stability, but the observed negative values of the parameters indicate an excess of rare mutations that may be due to recent population growth. The median-joining network and the BAPS results for mtDNA, together with the fact that it was found only in two individuals sampled close to the border with France, suggest that the divergent haplotype H12 is the result of a recent dispersal from France, and unrelated to the evolutionary history of the stone marten in Iberia since their first arrival in the peninsula. Other than this episode, the observed mtDNA diversity is consistent with a single colonization event at the origin of the stone marten population in the Iberian Peninsula.

The BSP analysis suggested a period of constant population size from 6,500 to 1,000 YBP, after which the Iberian population experienced a demographic expansion. These estimates, given the absence of suitable calibration points, depend on the estimated substitution rate but agree remarkably well with the age of the oldest fossils of stone marten in the Iberian Peninsula, dating to 3,000–5,000 YBP in the north [32] and 7,000 YBP in the south [32,33]. In this context, it would be important to derive estimates of the time of the first migration of stone martens into Iberia based on their genetic divergence from other populations in Europe, particularly those closest to the Iberian Peninsula (currently underway). Also, because colonization from France across the Pyrenees is not the only possible scenario, since the stone marten could have been introduced from elsewhere in the Mediterranean range of the species [32–34].

In any case, the apparent recent colonization of the Iberian Peninsula could explain the lack of phylogeographic structure found in this study, in contrast to what was observed in the other marten species present in Iberia, the pine marten *Martes martes* [93]. Our results also support a single taxonomic unit in the Iberian Peninsula, in agreement with [41]. The purported differences in size and coloration leading to the recognition of two subspecies in Iberia, the nominal subspecies *M*. *foina foina* (Erxleben, 1777) in the northern half of the peninsula and *M*. *foina mediterranea* (Barrett-Hamilton, 1898) in the south, likely fall within the range of continuous morphological variation displayed by the species in the region.

The phylogeographic signal for the stone marten expansion across Iberia, after its putative arrival in NE Spain from France, is still visible in the spatial patterns of genetic diversity. Haplotype richness was highest in NE Spain and there was a clear east-west differentiation in haplotype frequencies (Fig 2). The Ebro River, the upper courses of the rivers Douro, Tagus and Guadiana, and the Meseta in central Iberia have all likely continuously contributed to these patterns. In line, we observed a marked NE-SW cline of microsatellite allele frequencies in the sPCA and GLSI analyses (Fig 4c and 4d).

### Population genetic structure

The different Bayesian clustering methods did not converge to the same solution, but they all indicated two distinct genetic units in NE Spain and south Portugal (Fig 3). Across methods, the cluster in south Portugal (Fig 3, in yellow) was the most strongly differentiated one, as evidenced by the highest values of pairwise Fst (0.061–0.137, S5 Table), as well as of D_EST_ and G''_ST_ (0.154 and 0.244, respectively; S6 Table). The individuals in this cluster had the highest membership coefficients in all IBC algorithms used (data not shown). The cluster was also supported by the substantial degree of genetic distinctiveness of the southern Portuguese samples revealed by the interpolation plot (Fig 4d). The cluster in NE Spain was less differentiated from the neighbouring cluster, as hinted by the smaller values of Fst (0.033), D_EST_ (0.049), and G''_ST_ (0.080) (S5 and S6 Tables). Accordingly, the program TESS, which is especially suited for the detection of recent migrants [21], identified putative migrants between these two clusters.

Comparing these findings with the pattern of mtDNA differentiation, it can be hypothesized that the increased microsatellite divergence of the clusters in NE Spain and south Portugal may be the result of the long-term barrier effect of the River Ebro and of the rivers Tagus and Guadiana, respectively, and the high mutation rate of microsatellites. The importance of major rivers as barriers to gene flow in mesocarnivores has been identified in previous studies on other species (e.g. badger, *Meles meles* [94]; wildcat, *Felis silvestris* [9]). Interestingly, [7] in their study of the genetic structure of the Eurasian otter *Lutra lutra*, also using spatial and non-spatial IBC methods, found a genetic break in the Iberian Peninsula roughly corresponding to the course of the Tagus river. Similarly, in our study, GENELAND suggested that the location of the boundary separating the cluster in NE Spain might not strictly correspond to the River Ebro, at variance with the results from BAPS and TESS (Fig 3). Although GENELAND is not an edge detection method itself, like for instance those based on Wombling [95] or on the Monmonier algorithm [80], it has been shown to consistently outperform both boundary detection methods and other Bayesian clustering methods in detecting barriers to gene flow [17,96]. The boundary suggested by GENELAND coincides with the convergence of the Ebro Depression and the Iberian System mountain range, which separates the Ebro basin from the watersheds of the major rivers in central Iberia. Hence, even the major rivers in the Iberian Peninsula may only represent moderate barriers to gene flow for the stone marten, but they certainly contribute to shape its genetic structure.

Other than the two consensus clusters in NE Spain and south Portugal, the methods disagreed on the grouping of the individuals elsewhere in between (Fig 3). This could be the result of IBD, as Bayesian clustering methods are known to be sensitive to IBD and may infer spurious clusters when applied over a large area in which IBD is present [1,97] (see next section).

Of the clusters suggested by the Bayesian methods, the DAPC could only identify the cluster in south Portugal, apparently because it was relatively highly differentiated. This idea is supported by the fact that the clusters from DAPC had higher pairwise Fst values than those observed between the clusters revealed by the Bayesian methods (S4 Table). However, except the cluster in south Portugal, all the other four clusters inferred by DAPC did not have geographic identity.

### Inferring genetic structure using clustering methods in the presence of IBD

In continuously distributed species, mating with proximal individuals leads to patterns of close relatedness at finer scales and extensive genetic gradients at larger scales [11]. Accordingly, Mantel tests of IBD were significant for the whole study area (r = 0.296, P <0.001; Fig 4a) and within each of the three clusters identified by GENELAND (S5 Table). The large genetic neighbourhood inferred by the spatial autocorrelation analysis (452 km) further underlined the extent and strength of the spatial structure. Therefore, we conclude that the difficulties and inconsistencies shown by the clustering methods in determining the genetic structure of the Iberian stone marten were due to the presence of strong and pervasive IBD.

This phenomenon has been described in several empirical (e.g. [1,11]) and simulation studies [17,96,98]. The clustering algorithms are known to vary in their ability to accurately delimit genetic units in the presence of IBD and clines (e.g. [1,13,23], either underestimating [25] or overestimating the true number of populations [1,11]. Besides the widespread IBD in the Iberian stone marten, other characteristics of this study likely increased the challenge. First, the large size of the study area entailed the presence of population processes at different hierarchical levels, which may confound clustering methods [3]. Second, the study design has been shown to influence the performance of spatially-explicit IBC algorithms [11,99,100]. In this study however, even if the sampling was opportunistic, the resulting sampling scheme approximated reasonably well the heterogeneous distribution of the stone marten in Spain [41] (UTM10x10 grids; Fig 1). Although widespread and locally abundant, the species is irregularly distributed through the Iberian Peninsula and rare in some areas with apparently suitable habitat [41,101], (Fig 1).

## Conclusions and Perspectives

The phylogeography of the stone marten in the Iberian Peninsula is compatible with a single recent colonization of the region, possibly in the early Holocene, followed by population expansion sometime later. In turn, its contemporary genetic structure is characterized by a significant pattern of IBD and an apparent differentiation of the populations at the extreme of an NE-SW axis. To our knowledge, this is the first phylogeographic and population genetic study of the stone marten at broad regional scale. This case study also shows the usefulness of comparing and integrating multiple different techniques in the analysis of spatial genetic structure of mobile and continuously distributed species. Comprehensive analyses and tests of isolation by distance and clinal variation are an essential complement to clustering methods aiming to identify distinct genetic units, in particular due to the difficulties of the latter approaches in dealing with populations under IBD.

The genetic breaks detected in the Iberian stone marten population seemed largely related to the major river basins in the peninsula. However, linear barriers are only one of the multiple ways of how landscapes can influence gene flow [17]. Therefore, future studies are needed that focus on the importance of landscape features and variables in determining genetic structure (e.g. [102,103]). These should incorporate explicit tests on the presence of isolation by barriers and isolation by landscape resistance (e.g. land cover, elevation and temperature), especially in the areas where genetic discontinuities were inferred in this study.

## Supporting Information

S1 Table Information on the 352 stone marten specimens analysed in this study. (XLSX)

S2 Table Properties of the 23 multiplexed microsatellite loci used in this study, including repeat type, dye type, allele size range, number of alleles (Na) and observed (Ho) and expected (He) heterozygosities for each locus. (DOCX)

S3 Table mtDNA summary statistics for the NW, NE, SW and SE regions of Iberia. (DOCX)

S4 Table Pairwise Fst values between the inferred clusters by each of the clustering methods. (DOCX)

S5 Table Genetic diversity indices for the three clusters identified by GENELAND. (DOCX)

S6 Table Matrix of pairwise values of Jost’s DEST (above diagonal) and Hedrick´s unbiased G''ST (below diagonal) between the clusters inferred by GENELAND. (DOCX)
