# Supplementary material for: Inferring Population Genetic Structure in Widely and Continuously Distributed Carnivores: The Stone Marten (Martes foina) as a Case Study
Source: PLoS One. 2015 Jul 29;10(7):e0134257. doi: 10.1371/journal.pone.0134257 (PMC4519273; doi:10.1371/journal.pone.0134257)
Supplement: S2 Table — (DOCX) [file pone.0134257.s002.docx]

**S2 Table. Properties of the 23 multiplexed microsatellite loci used in this study, including repeat type, dye type, allele size range, number of alleles (Na) and observed (Ho) and expected (He) heterozygosities for each locus.**

| **Multiplex** | **Locus** | **Repeat** | **DYE** | **Size range** | **Na** | **Ho** | **He** |
| --- | --- | --- | --- | --- | --- | --- | --- |
| Multiplex A | Mlut27 | dinucleotide | PET | 178-202 | 8 | 0.341 | 0.375 |
|  | Mel1 | dinucleotide | PET | 264-276 | 6 | 0.468 | 0.577 |
|  | Mf1.1 | tetranucleotide | VIC | 152-168 | 5 | 0.399 | 0.446 |
|  | Mf4.17 | tetranucleotide | 6-FAM | 194-242 | 13 | 0.773 | 0.854 |
|  | Mf8.10 | tetranucleotide | NED | 128-152 | 7 | 0.648 | 0.738 |
| Multiplex B | Lut453 | dinucleotide | 6-FAM | 104-110 | 4 | 0.398 | 0.415 |
|  | Ma1 | dinucleotide | PET | 205-215 | 6 | 0.644 | 0.689 |
|  | Mf1.18 | tetranucleotide | PET | 153-165 | 4 | 0.374 | 0.391 |
|  | Mf3.7 | tetranucleotide | VIC | 181-201 | 6 | 0.398 | 0.456 |
|  | Mf8.8 | tetranucleotide | 6-FAM | 223-255 | 9 | 0.681 | 0.799 |
|  | Mp0059 | dinucleotide | VIC | 142-152 | 6 | 0.497 | 0.557 |
| Multiplex C | Lut615 | dinucleotide | 6-FAM | 115-127 | 6 | 0.301 | 0.47 |
|  | Ma2 | dinucleotide | PET | 174-186 | 6 | 0.437 | 0.491 |
|  | Mf1.11 | tetranucleotide | NED | 205-217 | 4 | 0.345 | 0.391 |
|  | Mf2.13 | tetranucleotide | 6-FAM | 285-297 | 4 | 0.388 | 0.588 |
|  | Mf3.2 | tetranucleotide | PET | 146-170 | 7 | 0.648 | 0.677 |
|  | Mf6.5 | tetranucleotide | VIC | 215-247 | 9 | 0.642 | 0.695 |
|  | Mvi072 | dinucleotide | NED | 262-276 | 8 | 0.351 | 0.436 |
| Multiplex D | Lut435 | dinucleotide | NED | 128-144 | 6 | 0.344 | 0.416 |
|  | Mf1.3 | tetranucleotide | NED | 196-220 | 7 | 0.591 | 0.645 |
|  | Mf4.10 | tetranucleotide | VIC | 311-339 | 8 | 0.649 | 0.769 |
|  | Mf8.7 | tetranucleotide | PET | 151-171 | 6 | 0.559 | 0.632 |
|  | Mvi-57 | dinucleotide | 6-FAM | 101-109 | 5 | 0.469 | 0.543 |
| **Mean** |  |  |  |  | **6.52** | **0.493** | **0.567** |
