# Supplementary material for: Inferring Population Genetic Structure in Widely and Continuously Distributed Carnivores: The Stone Marten (Martes foina) as a Case Study
Source: PLoS One. 2015 Jul 29;10(7):e0134257. doi: 10.1371/journal.pone.0134257 (PMC4519273; doi:10.1371/journal.pone.0134257)
Supplement: S3 Table — (DOCX) [file pone.0134257.s003.docx]

**S3 Table**. **mtDNA summary statistics for the NW, NE, SW and SE regions of Iberia**. Number of individuals (n) and haplotypes (Nh), private haplotypes (Ph), nucleotide diversity (π), haplotype diversity (H), and haplotypic richness (Hr) calculated using the rarefaction method to correct for unequal sample sizes (n=21-108; rarefaction to 15).

| **Region** | **n** | **Nh** | **Ph** | **π** | **H** | **Hr** |
| --- | --- | --- | --- | --- | --- | --- |
| NW | 54 | 5 | 1 | 0.00080 | 0.4934 | 2.374 |
| NE | 108 | 9 | 4 | 0.00131 | 0.7542 | 3.990 |
| SW | 69 | 5 | 0 | 0.00099 | 0.5550 | 1.997 |
| SE | 21 | 5 | 1 | 0.00166 | 0.7480 | 3.628 |
