# Supplementary material for: Inferring Population Genetic Structure in Widely and Continuously Distributed Carnivores: The Stone Marten (Martes foina) as a Case Study
Source: PLoS One. 2015 Jul 29;10(7):e0134257. doi: 10.1371/journal.pone.0134257 (PMC4519273; doi:10.1371/journal.pone.0134257)
Supplement: S4 Table — (DOCX) [file pone.0134257.s004.docx]

**S4 Table. Pairwise Fst values between the inferred clusters by each of the clustering methods.** All values were significantly different from zero at p<0.001.

| **DAPC** |  | DAPC_red | DAPC_purple | DAPC_orange | DAPC_yellow |
| --- | --- | --- | --- | --- | --- |
| DAPC_green (n:62) |  | 0.0887 | 0.0711 | 0.0624 | 0.1372 |
| DAPC_red (n:78) |  | - | 0.0541 | 0.0611 | 0.0897 |
| DAPC_purple (n:72) |  |  | - | 0.0604 | 0.078 |
| DAPC_orange (n:53) |  |  |  | - | 0.1191 |
| DAPC_yellow (n:68) |  |  |  |  | - |
| **STRUCTURE** |  | STR_red | STR_purple | STR_yellow |  |
| STR_green (n:106) |  | 0.0355 | 0.0625 | 0.1129 |  |
| STR_red (n:104) |  | - | 0.0348 | 0.1043 |  |
| STR_purple (n:68) |  |  | - | 0.0621 |  |
| STR_yellow (n:55) |  |  |  | - |  |
| **BAPS** |  | BAPS_red | BAPS_purple | BAPS_yellow |  |
| BAPS_green (n:121) |  | 0.0555 | 0.041 | 0.1045 |  |
| BAPS_red (n:58) |  | - | 0.0437 | 0.0846 |  |
| BAPS_purple (n:96) |  |  | - | 0.0957 |  |
| BAPS_yellow (n:58) |  |  |  | - |  |
| **TESS** |  | TESS_red | TESS_purple | TESS_yellow |  |
| TESS_green (n:111) |  | 0.0408 | 0.0657 | 0.0977 |  |
| TESS_red (n:127) |  | - | 0.0545 | 0.0783 |  |
| TESS_purple (n:35) |  |  | - | 0.061 |  |
| TESS_yellow (n:60) |  |  |  | - |  |
| **GENELAND** |  | GL_red | GL_yellow |  |  |
| GL_green (n:150) |  | 0.0330 | 0.1024 |  |  |
| GL_red (n:126) |  | - | 0.0753 |  |  |
| GL_yellow (n:57) |  |  | - |  |  |
