# Supplementary material for: Inferring Population Genetic Structure in Widely and Continuously Distributed Carnivores: The Stone Marten (Martes foina) as a Case Study
Source: PLoS One. 2015 Jul 29;10(7):e0134257. doi: 10.1371/journal.pone.0134257 (PMC4519273; doi:10.1371/journal.pone.0134257)
Supplement: S5 Table — (DOCX) [file pone.0134257.s005.docx]

**S5 Table**. **Genetic diversity indices for the three clusters identified by GENELAND**. Number of individuals (n), mean number of alleles (A), private alleles (Pa) observed (Ho) and expected (He) heterozygosities, overall Fis, Hardy-Weinberg equilibrium p-value, and Mantel’s correlation (r) of IBD tests, p<0.001).

| **Cluster** | **n** | **A** | **Pa** | **Ho** | **He** | **Fis** | **HWE** | **Mantel’s r** |
| --- | --- | --- | --- | --- | --- | --- | --- | --- |
| GL_red | 126 | 5.56 | 14 | 0.49 | 0.57 | 0.152 | <0.001 | 0.227 |
| GL_green | 150 | 5.78 | 19 | 0.53 | 0.6 | 0.129 | <0.001 | 0.145 |
| GL_yellow | 57 | 3.82 | 0 | 0.47 | 0.52 | 0.117 | <0.001 | 0.272 |
| total | 333 | 6.6 | 33 | 0.49 | 0.6 | 0.167 | <0.001 | 0.295 |
