# Supplementary material for: Inferring Population Genetic Structure in Widely and Continuously Distributed Carnivores: The Stone Marten (Martes foina) as a Case Study
Source: PLoS One. 2015 Jul 29;10(7):e0134257. doi: 10.1371/journal.pone.0134257 (PMC4519273; doi:10.1371/journal.pone.0134257)
Supplement: S6 Table — (DOCX) [file pone.0134257.s006.docx]

**S6 Table. Matrix of pairwise values of Jost’s D_EST_ (above diagonal) and Hedrick´s unbiased G''_ST_ (below diagonal) between the clusters inferred by GENELAND**. All values were significantly different from zero at p<0.001 (9999 permutations).

| **G''_ST_ \D_EST_** | GL_green | GL_red | GL_yellow |
| --- | --- | --- | --- |
| GL_green (n:150) | - | 0.049 | 0.100 |
| GL_red (n:126) | 0.080 | - | 0.154 |
| GL_yellow (n:57) | 0.169 | 0.244 | - |
